# PET Imaging of CD38 and IND enabling studies of [^89^Zr]Zr-DFO-Isatuximab

**DOI:** 10.1007/s11307-025-02062-9

**Published:** 2025-12-09

**Authors:** Brian D. Wright, Hailey A. Houson, Solana Fernandez, Kadir Gultekin, Jonathan E. McConathy, Smith Giri, Suzanne E. Lapi

**Affiliations:** 1 Department of Radiology, University of Alabama at Birmingham, Birmingham, AL 35294 USA; 2 Department of Biomedical Engineering, University of Alabama at Birmingham, Birmingham, AL 35294 USA; 3 Department of Medicine, University of Alabama at Birmingham, Birmingham, AL 35294 USA

**Keywords:** Zirconium-89, Isatuximab, CD38, Dosimetry

## Abstract

**Background:**

CD38 is an excellent biomarker and therapeutic target for multiple myeloma due to its high expression on cancerous cells in comparison to healthy cells.

**Purpose:**

We aimed to adapt Isatuximab as a PET imaging agent to detect CD38 positive multiple myeloma.

**Methods:**

*In vitro* studies confirmed the specificity of [^89^Zr]Zr-DFO-Isatuximab in CD38 + OPM-2 and MM.1S cells. Upregulation of CD38 was performed using pomalidomide and ricolinostat. Athymic nude mice were implanted with OPM-2 tumors and PET/CT images were collected 24 h, 3d, and 7d post-injection. Dosimetry data was collected from male and female mice and calculated using OLINDA. Three productions of [^89^Zr]Zr-DFO-Isatuximab were produced using GMP techniques and validated for use in the clinic.

**Results:**

Upregulation of CD38 was observed *in vitro* in CD38 + cells when treated with either pomalidomide or ricolinostat. *In vivo* evaluation of [^89^Zr]Zr-DFO-Isatuximab showed high selectivity in OPM-2 xenografts. Blocking with an excess of unlabeled Isatuximab reduced the tumor accumulation of [^89^Zr]Zr-DFO-Isatuximab by 45.5–48.5% confirming the *in vivo* specificity of this radiotracer. Dosimetry calculations were performed and showed an estimated effective dose of 0.359 mSv/MBq in females and 0.327 mSv/MBq in males. Three clinical grade [^89^Zr]Zr-DFO-Isatuximab doses using good manufacturing practices were synthesized which passed all quality control requirements and were stable up to 6 h, thus validating this compound for use in future clinical trials.

**Conclusion:**

[^89^Zr]Zr-DFO-Isatuximab showed high specificity to CD38 positive cells, had estimated effective doses comparable to other clinically relevant ^89^Zr-labeled antibodies, and can be prepared using GMP practices for clinical use.

**Supplementary Information:**

The online version contains supplementary material available at 10.1007/s11307-025-02062-9.

## Introduction

Multiple myeloma is a rare disease that comprises 1% of all cancers worldwide. It is the second most common hematologic malignancy after lymphoma and is associated with substantial morbidity and mortality [1–3]. Current methods of detection such as blood analysis or bone marrow biopsy have room for improvement as not all multiple myeloma secrete the abnormal immunoglobulins required for blood analysis and biopsies are invasive and may inadequately capture the clonal heterogeneity associated with this disease [4–6]. Thus a non-invasive technique to detect myeloma and confirm the efficacy of targeted treatment is needed.

CD38 is a transmembrane glycoprotein that is highly expressed by multiple myeloma cells and plays a role in activation and proliferation signals [7]. Isatuximab is a monoclonal antibody (mAb) that binds to the Met110 to Cys119 epitope on the human CD38 receptor [8]. Preclinical data has revealed Isatuximab to have anti-myeloma activity with multiple mechanisms of action [9, 10]. Reports have shown high expressing CD38 cell lines treated with Isatuximab can go through complement-dependent cytotoxicity, antibody-dependent cellular phagocytosis (ADCP) and antibody-dependent cellular cytotoxicity (ADCC), whereas low expressing CD38 cell lines only go through ADCC [9]. Promising efficacy was observed in phase III clinical trials which led to the FDA approval of the use of Isatuximab in patients with relapsed refractory multiple myeloma in 2020 [10, 11]. As the efficacy of this and other CD38 targeted monoclonal antibody treatments has been linked to CD38 expression levels, it is important to evaluate CD38 levels in patients [11]. Expression of CD38 can be measured through a series of bone marrow biopsies, however this approach may not accurately report the heterogeneity seen across different sites of disease [6]. To develop a less invasive, more accurate test we aimed to investigate a non-invasive imaging technique for CD38 using radiolabeled Isatuximab.

Positron emission tomography (PET) is a highly sensitive technique that can be used to track antibody pharmacokinetics non-invasively. A variety of mAbs have been explored in the literature and shown to have excellent targeting specificity while maintaining the sensitivity of PET [12–15]. ^89^Zr is a positron emitting radionuclide with a half-life of 3.3 days that is well matched with the pharmacokinetic properties of intact antibodies and antibodies radiolabeled with ^89^Zr have been tracked non-invasively *in vivo* for over 7 days in both preclinical and clinical subjects [12–14].

Previous studies have shown that both [^89^Zr]Zr-DFO-Isatuximab and [^89^Zr]Zr-DFO-Daratumumab are suitable for imaging of CD38 in preclinical models with high sensitivity and specificity with [^89^Zr]Zr-DFO-Daratumumab translated into human studies [16–19]. These agents bind to different epitopes and could be used in a complementary fashion as it may be possible to image patients undergoing treatment with either Isatuximab or Daratumumab with the other labeled antibody to monitor progress without worry of competition for binding sites. Some reports have mentioned Isatuximab showing efficacy in Daratumumab refractory cancers [20]. Studies have also shown expression of CD38 can be increased through the treatment of cells with pomalidomide or ricolinostat and have shown to increase the efficacy of both Daratumumab and Isatuximab [3, 10, 20–22]. To further develop CD38 imaging techniques, we aimed to assess the imaging characteristics of [^89^Zr]Zr-DFO-Isatuximab in preclinical animal models, evaluate the effects of CD38 upregulation on Isatuximab *in vitro*, conduct dosimetry studies to enable the translation to human use and to develop procedures compatible with GMP operations. *In vitro* studies were also carried out to assess the feasibility of drug induced upregulation of the CD38 receptor.

## Methods

### Reagents and Cell Cultures

Isatuxumab was obtained from Sanofi (Paris, France). Deferoxamine-p-benzyl-isothiocyanate (DFO) was purchased from Macrocyclics (Dallas, TX). All other chemicals were purchased from Fisher Scientific (Hampton, NH). The low CD38 expressing U266 and the CD38 expressing multiple myeloma cell line OPM-2 was purchased from the Leibniz Institute German Collection of Microorganisms and Cell Cultures (DSMZ, Brunswick, Germany). The CD38 expressing MM.1S multiple myeloma cell lines were purchased from the American Type Culture Collection (ATCC, Manasas, VA) [23]. Cells were cultured in ATCC modified RPMI 1640 containing 20% fetal bovine serum (FBS) in a humidified incubator with 5% CO_2_ at 37 °C. Reagents for cell culture were purchased from Fisher Scientific.

### Antibody Conjugation and Radiolabeling

A solution of 20 mg/mL of Isatuximab was adjusted to pH 9 using 0.1 M sodium carbonate buffer and combined with 16 molar equivalents of deferoxamine-Bz-SCN (DFO) in DMSO. The resulting mixture was incubated at 37 °C for 1 h with agitation. After incubation the unreacted DFO was removed using a 40 k MWCO Zeba spin column which also served to buffer exchange the conjugate into 1 M HEPES.

Zirconium-89 was produced in the University of Alabama at Birmingham cyclotron facility as zirconium oxalate, diluted with 1 M HEPES buffer and neutralized with 2 M sodium hydroxide. Solutions of 222 MBq/mg (6 mCi/mg) of the neutralized zirconium-89 and Isatuximab-DFO solution were combined and incubated for 1 h at 37 °C with agitation. After incubation, a sample of the labeled antibody was challenged with a 50 mM DTPA solution, and the radiochemical purity was assayed via iTLC using a 50 mM DTPA solution as the running buffer.

### Stability Studies

Radiolabeled [^89^Zr]Zr-DFO-Isatuximab was combined with saline, mouse serum, or human serum and incubated at 37 °C for 7 days. Samples were collected at 0 h, 1 d, 2 d, 3 d, and 7 d and analyzed via iTLC using a running buffer of 50 mM DTPA.

### *In Vitro* Cell Studies

The Lindmo assay was used to determine the *in vitro* binding characteristics of [^89^Zr]Zr-DFO-Isatuximab [24]. Concentrations ranging from 0.5 to 5 × 10^6^ cells/mL of OPM-2 (CD38 +) cells along with a blank were combined with 50 µL of a 1% BSA solution containing 185 Bq (5 nCi, 1 ng) of the radiolabeled antibody in microcentrifuge tubes and diluted to a total volume of 550 µL with PBS. The cells were allowed to incubate at room temperature with gentle agitation for 1 h, pelleted, and washed with cold PBS three times. The total amount of activity was counted on a Perkin Elmer gamma counter, the results from the blank were subtracted from all values, and the binding was calculated as the ratio of bound activity to the total amount of activity.

CD38 upregulation assays were performed based on previous methods [21, 22]. OPM-2 (CD38 +), U-266 (CD38-), and MM.1S (CD38 +) multiple myeloma cells were cultured in RPMI-1640 with 20% fetal bovine serum at 3 × 10^5^ cells/well in 24-well flat bottom plates. Pomalidomide or Ricolinostat was reconstituted in dimethyl sulfoxide and added to the medium to reach a final concentration of 0, 1, 5, or 10 µM. After 72 h, the cells were exposed to 1 ng of [^89^Zr]Zr-DFO-Isatuximab (148 Bq, 4 nCi) for 1 h at room temperature with gentle agitation. After incubation, the cells were collected, washed with cold PBS three times, and assayed for radioactivity. Protein concentration in each well was determined via the BCA assay and the binding was expressed as the % bound activity/ug of protein.

### *In Vivo* Studies

All animal experiments were conducted according to the guidelines of the Institutional Animal Care and Use Committee (IACUC) and approved by the University of Alabama at Birmingham Animal Studies Committee. All PET imaging and biodistribution studies were performed using athymic nude mice from Charles River Laboratories. Mice were inoculated in the shoulder with 100 µL of 4 × 10^6^ cells/mL in Matrigel. Tumors reached 5 mm in the greatest dimension after 30 days with a 70% take rate. Once tumors were palpable, the mice were separated into two groups and injected via tail-vein with either 100 µL of 3.5 MBq (25 µg, 100 µCi) of [^89^Zr]Zr-DFO-Isatuximab or 100 µL of 3.5 MBq (25 mg, 100 µCi) of [^89^Zr]Zr-DFO-Isatuximab with a 1 mg Isatuximab blocking dose in saline. PET (energy window 350–650 keV) and CT (voltage 80 kVp, current 150 µA, 720 projections, scan time 5 min) images were acquired using a GNEXT small animal PET/CT scanner (Sofie Biosciences, Dulles, VA) at 1, 3, and 7 days after injection. After 24 h and 7 days post injection mice were euthanized, and organs were harvested, weighed, and assayed in the gamma counter for biodistribution studies. The PET images were reconstructed using a 3D-OSEM (Ordered Subset Expectation Maximization) algorithm (24 subsets and 3 iterations) with random, attenuation, and decay correction. The CT images were reconstructed using a Modified Feldkamp Algorithm. Image analysis was performed using the Vivoquant software suite (Invicro, Needham, MA). Regions of interest (ROI) were drawn for tumor uptake and analyzed as standard uptake values (SUV) using the formula SUV = (MBq/mL) x (animal weight (g)/injected dose (MBq)). Radioactivity associated with each organ was expressed as a percentage of the injected dose per gram of organ (%ID/g).

### *In Vivo* Dosimetry Studies

Groups of male or female mice (*n* = 5) were injected via tail-vein with 100 µL of 3.5 MBq (25 µg, 100 µCi) of [^89^Zr]Zr-DFO-Isatuximab in saline. Mice were euthanized at 1 h, 4 h, 24 h, 2 d, 5 d, and 7 d post injection and organs were harvested, weighed, and assayed in the gamma counter for biodistribution studies. Separate groups of male and female animals were injected and placed into metabolic cages for 24 h to access urine and feces excretion. Radioactivity associated with each organ was expressed as a percentage of the injected dose per organ (%ID/organ).

### Dosimetry Analysis

Radioactivity amounts from each time point for each organ were utilized to calculate time-integrated activity coefficients (TIACs) [25]. Area under curve (AUC) for TIACs was calculated using Python programming tools with libraries including Pandas for data manipulation, Numpy for numerical calculations, and Matplotlib for graphical representation. The AUC for each organ’s activity over time was determined using numerical integration, applying Simpson`s Rule for the majority of the data points and the Trapezoidal rule for the last segment if an odd number of intervals were present. From the last observed data points to infinity, physical decay was assumed for ^89^Zr to extrapolate activity to infinity.

Extrapolation of human absorbed dose from mouse biodistribution experiments was calculated with direct mice-to-human extrapolation [25].

$$TIAC, h (organ) = TIAC, m (organ)$$

The MIRD schema for internal dosimetry was used to calculate the absorbed-dose coefficients and contribution to effective doses per organ [26]. A generic equation for the absorbed dose in any target organ can be found below from the Olinda program.

$$D = k\frac{\widehat{A}{\sum }_{i}{n}_{i}{E}_{i}{\phi }_{i}}{m}$$

where:

D = absorbed dose in a target organ (rad or Gy).

Ã = cumulated activity (sum of all nuclear transitions (disintegrations) that occurred) in a source organ (μCi/hr or MBq-s).

*n* = number of radiations with energy E emitted per nuclear transition.

E = energy per radiation (MeV).

ϕ= absorbed fraction (fraction of radiation energy absorbed in the target).

m = mass of target region (g or kg).

k=proportionality constant (rad-g/μCi-hr-MeV or Gy-kg/MBq-sMeV).

### Statistical Analysis

Statistical analysis was performed using Prism Software (GraphPad, San Diego, CA). Unpaired Student’s t-tests were used to analyze the SUV and biodistribution data. Differences with a p-value of < 0.05 were considered statistically significant.

### Preparation of GMP Grade [^89^Zr]Zr-DFO-Isatuximab

All processes were performed using aseptic techniques in a clean ISO-5 laminar flow hood. 100 mg of Isatuximab (20 mg/mL, 5 mL) was combined with 4.5 mL of 0.1 M sodium carbonate to adjust the pH to 9. After 10 min, 16 molar equivalents of deferoxamine-Bz-SCN (DFO) in 0.5 mL of DMSO was added and the resulting mixture was incubated at 37 °C for 1 h with agitation. After incubation the unreacted DFO was removed using a 40 k MWCO Zeba spin column which also served to buffer exchange the conjugate into 1 M HEPES. Following purification the antibody-DFO conjugate was aliquoted into 0.5 mL (5 mg) samples, and the BCA assay was used to determine the concentration of the batch.

In a freshly cleaned laminar flow hood 1 vial of GMP grade DFO-Isatuximab (0.5 mL, 5 mg) was combined with 370 MBq (10 mCi) of neutralized zirconium-89. The mixture was incubated at 37 °C for 1.5 h with agitation after which the reaction was quenched with 50 µL of a 50 mM DTPA solution. The mixture was incubated at 37 °C for 5 min and then purified using a 40 k MWCO Zeba spin column which also served to buffer exchange the [^89^Zr]Zr-DFO-Isatuximab into 0.9% saline. The resulting labeled antibody solution was then filtered with a 0.2 µm Pall syringe filter into a sterile 30 mL vial and diluted to 10 mL using 0.9% saline.

### Quality Control of GMP Grade [^89^Zr]Zr-DFO-Isatuximab

#### Filter Membrane Integrity

After synthesis, the used filter was connected to a compressed air line, and the pressure slowly increased until the filter burst. The acceptance criteria was ≥ 46 psi and was based on manufacturer specifications.

#### Radiochemical Purity, Identity, and Protein Aggregation

Size exclusion HPLC was performed using a TOSOH TSKgel SuperSW3000 column and a running buffer of 50 mM sodium phosphate buffer (pH 7) with 150 mM sodium chloride and 0.1% Tween-20. The final product was compared to an unlabeled sample of Isatuximab in 0.9% saline as a standard. The final product radio peak was required to be within 10% of the UV peak of the standard.

#### Specific Activity

Preclinical work determined via the BCA assay that 10% of the labeled antibody was lost upon sterile filtration. This loss was included in the calculation for the specific activity equal to final product yield/4.5 mg.

#### Bacterial Endotoxin Levels

A 1:100 dilution was performed on the sterile filtered final product using sterile water for irrigation. This sample was analyzed with a Endosafe Nexgen PTS reader using a PTS cartridge with a sensitivity of 0.05 EU/mL. The acceptance criteria of 175 EU/V were derived from USP < 85 > guidelines for radiopharmaceuticals [27].

#### Sterility

In a freshly cleaned ISO-5 environment, 0.2 mL of [^89^Zr]Zr-DFO-Isatuximab was injected into Tryptic Soy Broth and Fluid Thioglycollate Medium. The vials were incubated at 20–25 °C or 30–35 °C respectively and monitored for 14 days post injection for growth. A result of no visible growth after 14 days indicated a passing result.

## Results

Deferoxamine-NCS was conjugated with Isatuximab in a 16:1 (mmol DFO:mmol Ab) ratio. The resulting antibody conjugate was optimized for labeling with ^89^Zr resulting in 100% radiochemical purity by iTLC at a maximum specific activity of 222 MBq/mg (6 mCi/mg, *n* = 8). This is significantly higher than previously reported specific activities of 33.3 MBq/mg and likely due to the larger amount of DFO conjugate used (4 molar equivalents vs 16 molar equivalents [16]. [^89^Zr]Zr-DFO-Isatuximab was found to be 100% stable in 0.9% saline, mouse serum, and human serum at 37 °C for 7 days *in vitro*. This stability matches the recent report by Herrero Alvarez et al.[16]. No decomplexation was observed via iTLC (100 mm) samples at any timepoint observed (Supplementary Figs. S1-S2).

### Cell Binding Studies of [^89^Zr]Zr-DFO-Isatuximab

Results of the Lindmo assay showed the immunoreactive fraction to be 97.4% indicating modifications to the antibody had not interfered with its ability to bind to CD38 on the OPM-2 cell line (Supplementary Fig. S3). The specificity of [^89^Zr]Zr-DFO-Isatuximab was investigated by comparing binding in moderate CD38 expressing cells (OPM-2) and low CD38 expressing cells (U-266). The CD38 expression of these cell lines have been previously characterized by flow cytometry by the manufacturer (DSMZ) as well as Ghose et al. and Drent et al. [23, 28] Cell binding of [^89^Zr]Zr-DFO-Isatuximab to the OPM-2 and U-266 cells was 9.9 ± 2.2% and 4.8 ± 1.0% respectively. Significantly greater binding was observed in the moderate CD38 expressing cells over the lower CD38 expressing cells (*p* = 0.04).

**Fig. 1:**
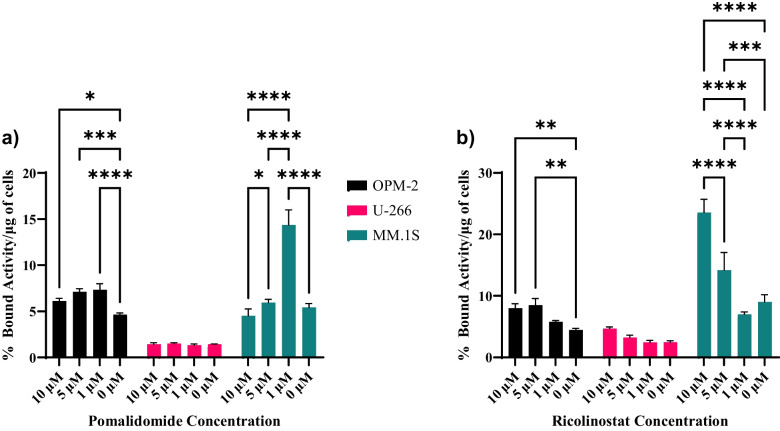
Cell binding and CD38 upregulation studies. **a** CD38 upregulation with pomalidomide. **b** CD38 upregulation with ricolinostat

OPM-2 and U-266, and MM.1S multiple myeloma cells were treated with Pomalidomide or Ricolinostat for three days and were incubated with [^89^Zr]Zr-DFO-Isatuximab. CD38 expression of the MM.1S cell line has been previously characterized by flow cytometry both Ghose et al. and Greenstein et al. [23, 29] Binding of the [^89^Zr]Zr-DFO-Isatuximab to the cells increased in both the OPM-2 and the MM.1S cells when exposed to the drug. Significantly greater binding was observed with the incubation with a 1 µM concentration of Pomalidomide (*p* < 0.0001, Fig. 1a). OPM-2 illustrated an increase from 4.6 ± 0.2% bound/µg in the control group to 7.3 ± 0.6% bound/ug in the 1 µM concentration group. MM.1S illustrated an increase from 5.4 ± 0.4% bound/µg in the control group to 14.4 ± 1.6% bound/ug in the 1 µM concentration group. Similarly, significantly greater binding was observed with the incubation with a 5 µM concentration of Ricolinostat in the OPM-2 cell line (*p* = 0.002, Fig. 1b) and with a 10 µM concentration in the MM.1S cell line (*p* < 0.0001, Fig. 1b). Neither Pomalidomide nor Ricolinostat had a significant effect on the binding of [^89^Zr]Zr-DFO-Isatuximab to the U-266 cell line.

### PET Imaging and Biodistribution of [^89^Zr]Zr-DFO-Isatuximab

**Fig. 2:**
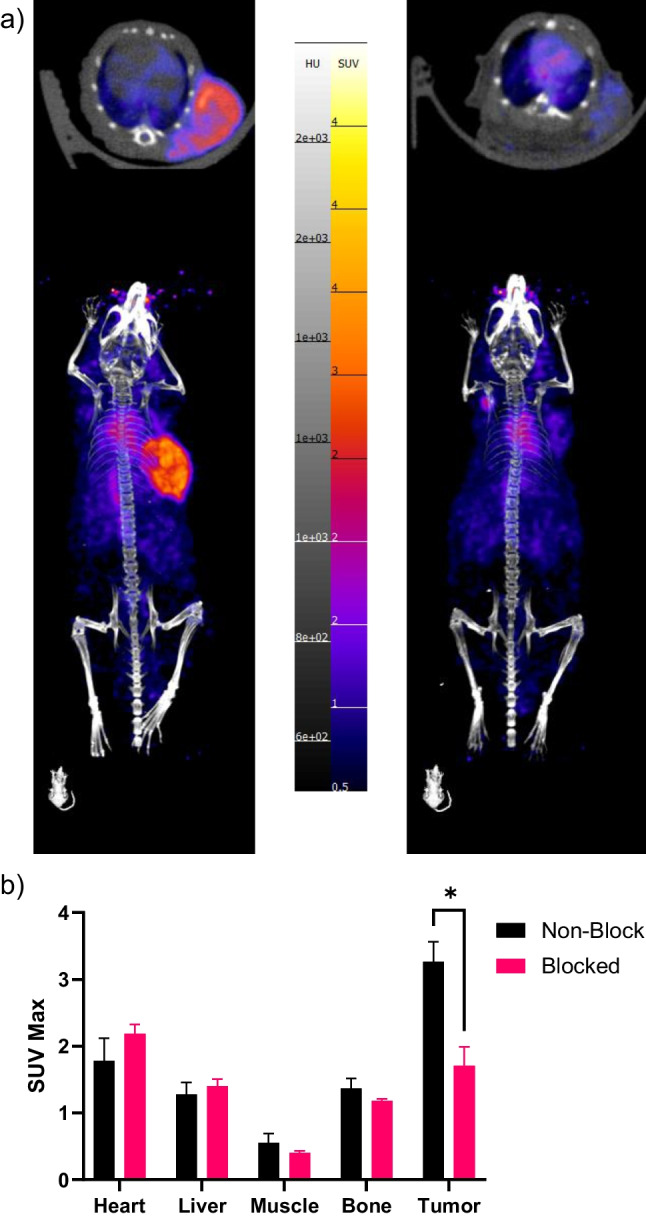
*In vivo* studies show high uptake of [^89^Zr]Zr-DFO-Isatuximab 3 d post-injection. **a** Maximum intensity projection (MIP) of PET/CT images of [^89^Zr]Zr-DFO-Isatuximab in OPM-2 tumors in the nonblocked (left) and blocked groups (right), **b** SUVMax analysis of [^89^Zr]Zr-DFO-Isatuximab

**Fig. 3:**
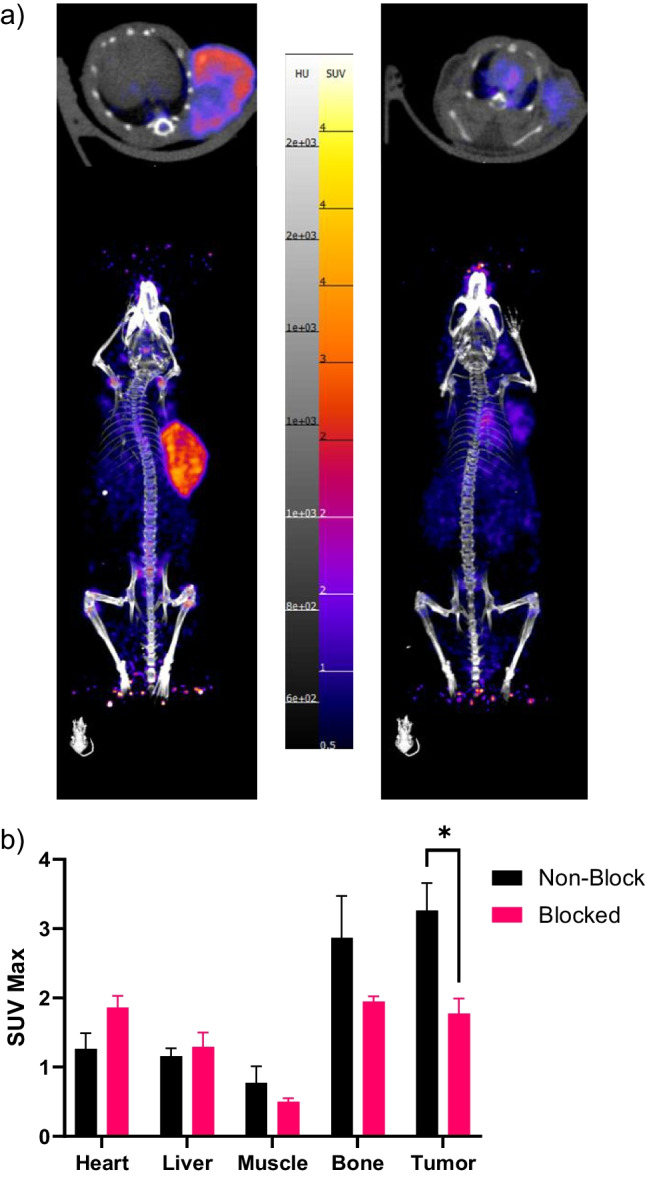
*In vivo* studies showing high uptake of [^89^Zr]Zr-DFO-Isatuximab 7 d post-injection. **a** Maximum intensity projection (MIP) of PET/CT images of [^89^Zr]Zr-DFO-Isatuximab in OPM-2 tumor bearing mice in the nonblocked (left) and blocked groups (right), **b** SUVMax analysis of [^89^Zr]Zr-DFO-Isatuximab

Athymic nude mice grew subcutaneous OPM-2 tumors 4 weeks after inoculation with take rates of 70% (14/20). PET imaging was performed 1 d, 3 d, and 7 d post injection of [^89^Zr]Zr-DFO-Isatuximab. One cohort was also co-injected with a blocking dose of non-radiolabeled Isatuximab. Accumulation in the OPM-2 tumor xenograft was higher in the [^89^Zr]Zr-DFO-Isatuximab group than that of the blocking group after three days with a max SUV of 3.3 ± 0.3 vs 1.7 ± 0.3 (*p* = 0.0029, Fig. 2). This trend continued out to seven days post injection with a SUV max of 3.3 ± 0.4 vs 1.8 ± 0.2 (*p* = 0.0099, Fig. 3). No significant differences were observed between the two groups in the heart, liver, muscle, or bone throughout the study.

**Fig. 4:**
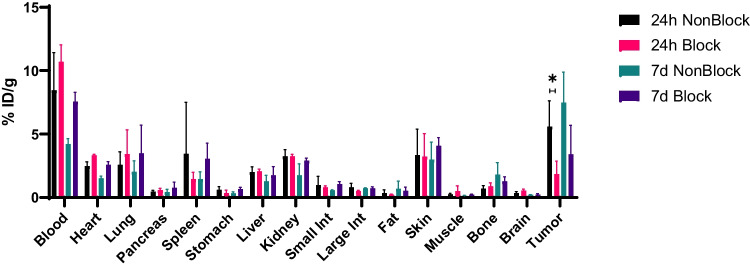
Biodistribution of [^89^Zr]Zr-DFO-Isatuximab in OPM-2 tumor bearing mice 24 h and 7 d post-injection in non-block and blocking groups

Biodistribution studies showed higher uptake in the tumors of the non-blocked group vs the blocked group at 24 h 5.6 ± 1.65 vs 1.8 ± 0.8%ID/g (*P* = 0.04, Fig. 4). This uptake increased over 6 days to 7.5 ± 2.0 vs 3.4 ± 1.9% ID/g (*P* > 0.05, Fig. 4). The difference in the day 7 post injection non-blocked group vs the blocked group, while not statistically significant, demonstrates the long biological half-life of Isatuximab and may be a factor in the high variance in tumors. Liver and kidney uptake were comparable to other antibodies in the literature suggesting similar clearance routes [12, 13].

### [^89^Zr]Zr-DFO-Isatuximab Dosimetry Analysis

The biodistribution and absorbed doses of [^89^Zr]Zr-DFO-Isatuximab were extrapolated to adult male and female subjects (*n* = 5 per timepoint) using TIACs derived from mice biodistribution measurements. In female animals, the left colon and kidneys demonstrated the highest absorbed-dose coefficients values of 1.17 mGy/MBq and 1.19 mGy/MBq, respectively, pointing to their roles in metabolic processing and clearance of the radiopharmaceutical. The ovaries (0.79 mGy/MBq) and liver (0.84 mGy/MBq) also received dose from this radiopharmaceutical (Table 1).

**Table 1:** Absorbed dose estimations from female mouse to human for [^89^Zr]Zr-DFO-Isatuximab

Organ Doses	Nuclide:(Zr)−89	ICRP 89	Adult Female	
Target organ	Beta (mGy/MBq)	Gamma (mGy/MBq)	Total (mGy/MBq)	ICRP-103-ED (mSv/MBq)
Adrenals	2.01E-01	5.44E-01	7.45E-01	6.88E-03
Brain	1.14E-02	8.48E-02	9.62E-02	9.62E-04
Breasts	1.54E-02	1.05E-01	1.20E-01	1.44E-02
Esophagus	1.60E-02	2.26E-01	2.42E-01	9.68E-03
Eyes	1.54E-02	9.27E-02	1.08E-01	0.00E + 00
Gallbladder wall	1.54E-02	4.33E-01	4.48E-01	4.14E-03
Left colon	6.62E-01	5.04E-01	1.17E + 00	5.66E-02
Small intestine	3.05E-01	4.20E-01	7.25E-01	6.69E-03
Stomach wall	7.91E-02	3.26E-01	4.06E-01	4.87E-02
Right colon	2.73E-01	4.38E-01	7.10E-01	3.45E-02
Rectum	4.71E-02	2.10E-01	2.57E-01	5.91E-03
Heart wall	1.80E-01	2.70E-01	4.50E-01	4.15E-03
Kidneys	5.07E-01	6.85E-01	1.19E + 00	1.10E-02
Liver	2.51E-01	5.90E-01	8.40E-01	3.36E-02
Lungs	4.45E-02	1.92E-01	2.36E-01	2.84E-02
Ovaries	4.33E-01	3.59E-01	7.92E-01	3.17E-02
Pancreas	7.56E-02	4.25E-01	5.01E-01	4.62E-03
Salivary glands	1.44E-01	1.53E-01	2.96E-01	2.96E-03
Red marrow	1.12E-02	2.00E-01	2.11E-01	2.53E-02
Osteogenic cells	1.93E-01	1.91E-01	3.84E-01	3.84E-03
Spleen	1.46E-01	3.95E-01	5.41E-01	4.99E-03
Thymus	2.97E-01	2.71E-01	5.67E-01	5.24E-03
Thyroid	1.54E-02	1.17E-01	1.32E-01	5.30E-03
Urinary bladder wall	2.66E-02	1.44E-01	1.70E-01	6.81E-03
Uterus	2.61E-01	3.79E-01	6.40E-01	2.96E-03
Total body	4.00E-02	1.66E-01	2.06E-01	0.00E + 00
Effective dose				3.59E-01

In male animals, the testes showed the highest absorbed-dose coefficients of 1.32 mGy/MBq. Similar to the female studies, the kidney dose was 1.16 mGy/MBq and the liver dose was 0.726 mGy/MBq. Doses of 0.402, 0.408, and 0.783 mGy/MBq were calculated for the right colon, the heart wall, and the spleen, respectively. (Table 2).

**Table 2:** Absorbed dose estimations from male mouse to human for [^89^Zr]Zr-DFO-Isatuximab

Organ Doses	Nuclide: Zr-89	ICRP 89	Adult Male	
Target organ	Beta (mGy/MBq)	Gamma (mGy/MBq)	Total (mGy/MBq)	ICRP-103-ED (mSv/MBq)
Adrenals	2.11E-01	5.75E-01	7.86E-01	7.26E-03
Brain	1.02E-02	8.87E-02	9.89E-02	9.89E-04
Esophagus	1.36E-02	2.09E-01	2.23E-01	8.91E-03
Eyes	1.35E-02	9.51E-02	1.09E-01	0.00E + 00
Gallbladder wall	1.67E-02	4.41E-01	4.57E-01	4.22E-03
Left colon	3.33E-01	3.70E-01	7.02E-01	3.41E-02
Small intestine	2.10E-01	3.54E-01	5.64E-01	5.20E-03
Stomach wall	6.59E-02	2.69E-01	3.35E-01	4.02E-02
Right colon	3.20E-01	4.62E-01	7.83E-01	3.80E-02
Rectum	4.32E-02	1.69E-01	2.13E-01	4.89E-03
Heart wall	1.30E-01	2.73E-01	4.02E-01	3.72E-03
Kidneys	5.03E-01	6.60E-01	1.16E + 00	1.07E-02
Liver	2.06E-01	5.20E-01	7.26E-01	2.90E-02
Lungs	5.68E-02	1.87E-01	2.44E-01	2.93E-02
Pancreas	6.19E-02	3.67E-01	4.29E-01	3.96E-03
Prostate	1.35E-02	1.38E-01	1.52E-01	7.01E-04
Salivary glands	1.56E-01	1.67E-01	3.22E-01	3.22E-03
Red marrow	9.99E-03	1.88E-01	1.98E-01	2.38E-02
Osteogenic cells	3.01E-01	2.02E-01	5.03E-01	5.03E-03
Spleen	1.13E-01	2.95E-01	4.08E-01	3.76E-03
Testes	8.11E-01	5.07E-01	1.32E + 00	5.27E-02
Thymus	2.98E-01	2.71E-01	5.70E-01	5.26E-03
Thyroid	1.35E-02	1.22E-01	1.35E-01	5.41E-03
Urinary bladder wall	2.82E-02	1.32E-01	1.60E-01	6.41E-03
Total body	3.70E-02	1.28E-01	1.65E-01	0.00E + 00
Effective dose				3.27E-01

The radiopharmaceutical's estimated effective dose of 0.359 mSv/MBq in females and 0.327 mSv/MBq in males falls within the range of similar diagnostic agents, supporting [^89^Zr]Zr-DFO-Isatuximab's safety profile in clinical diagnostics [14, 30, 31]. Studies from Laban, Zakaly, and La Forest all report effective doses around 0.5 mSv/MBq, which is slightly higher than what was observed in this study. Our results show a consistent pattern in the radiobiological safety profile across different ^89^Zr-labeled antibodies used in imaging applications.

### Human Use Dose Preparation and Stability

Clinical grade [^89^Zr]Zr-DFO-Isatuximab was produced using aseptic techniques and the SOPs for the synthesis were developed. Each batch was prepared starting with 370 MBq (10 mCi) of Zirconium-89 and yielded between 211–252 MBq (5.7–6.8) mCi of [^89^Zr]Zr-DFO-Isatuximab. While this results in a lower specific activity than the preclinical work, 74 MBq/mg vs 222 MBq/mg (2 mCi/mg vs 6 mCi/mg) it does ensure 100% labeling up to 78 h after the production of the [^89^Zr]Zr-oxalate. Three validation batches were synthesized and passed a set of predetermined quality control criteria (Table 3). The final products were clear and colorless without particles in the solution, and all filter membranes were intact after synthesis. HPLC analysis showed the final products were 100% radiochemically pure and labeling was confirmed by the comparison of the radioactive peak associated with the main UV peak to a nonlabelled DFO-Isatuximab standard. There was minimal protein aggregation observed in the final product with each of the three batches having the monomer UV peak ≥ 80% of the total UV peaks. The specific activity of the final products was found to be > 0.2 mCi/mg. No endotoxins were detected after synthesis, and the final products were found to be sterile after 14 days.

**Table 3:** [^89^Zr]Zr-DFO-Isatuximab validation batch quality control results

Quality Control Test	Analytical Method	Acceptance Criteria	Results Batch# 240,520-ISA-01	Results Batch# 240,621-ISA-01	Results Batch# 240,723-ISA-01
Filter membrane integrity	Bubble point test	≥ 46 psi	65.6 psi	59.9 psi	59.9 psi
pH	Narrow range pH test trips	4–6	5.0	5.5	5.5
Strength (radioactivity concentration)	Dose calibrator assay divided by volume	0.2–1.0 mCi/ml	0.57 mCi/ml	0.68 mCi/ml	0.65 mCi/ml
Appearance/color	Visual inspection for color and particulates	Clear, colorless, particulate-free	Clear, colorless, particulate-free	Clear, colorless, particulate-free	Clear, colorless, particulate-free
Radionuclidic purity	Gamma spectroscopy	≥ 99.5%	100%	100%	100%
Radionuclidic identity	Gamma spectroscopy	Identify major photopeak	511 & 909 keV Identified	511 & 909 keV identified	511 & 909 keV identified
Radiochemical purity	HPLC	> 95%	100%	100%	100%
Radiochemical identity	HPLC	Isatuximab and monomer peak retention times must agree by ± 10%	Rt Isatuximab std: 11.59 Rt monomer: 12.41 Pass	Rt Isatuximab std: 11.82 Rt monomer: 12.20 Pass	Rt Isatuximab std: 11.55 Rt monomer: 12.18 Pass
Specific activity	Dose calibrator assay divided by protein mass	≥ 0.2 mCi/mg	1.27 mCi/mg	1.50 mCi/mg	1.44 mCi/mg
Bacterial endotoxin levels	Rapid photometric method	≤ 175 EU/V (where V is the maximum total dose)	< 5 EU/ml Pass	< 5 EU/ml Pass	< 5 EU/ml Pass
Radioimmunoassay	Immunoreactivity assay	≥ 65%	100%	100%	100%
Protein aggregation	HPLC	≥ 80% monomer	100%	100%	92.9%
Sterility	Visual observation	No visible growth observed after 14 days	No visible growth observed after 14 days	No visible growth observed after 14 days	No visible growth observed after 14 days

For stability testing, the final drug product was prepared starting with 370 MBq (10 mCi) of Zirconium-89, stored inverted in the same container as it was produced in, and left at room temperature for up to 8 h. The expiration time of the final product was found to be 6 h after end of synthesis using analytical HPLC, pH, and a visual appearance evaluation (Table 4). This length of stability is acceptable and allows for the use of [^89^Zr]Zr-DFO-Isatuximab in the clinic on the day of production.

**Table 4:** Stability results for [^89^Zr]Zr-DFO-Isatuximab validation batches

Test	Acceptance Criteria [^89^Zr]-ISA	Test Time	Validation #1 (Results after 6, and 8 h)	Validation #2 (Results after 6, and 8 h)	Validation #3 (Results after 6 h)
[^89^Zr]Isatuximab Batch #	N/A	N/A	240,520-ISA-01	240,621-ISA-01	240,723-ISA-01
EOS time	N/A	N/A	13:08	10:29	15:05
Stability test time	N/A	6 h	19:19	16:29	21:05
		8 h	21:19	18:29	N/A
Protein aggregation	≥ 80% monomer	6 h	99.3%	91.2%	92.8%
		8 h	98%	95.2%	N/A
Radiochemical purity (HPLC):	Purity ≥ 95%	6 h	95.7%	100%	97.1%
		8 h	95.6%	89.3%	N/A
pH:	4.5–8.0	6 h	5.5	5.5	5.5
		8 h	5.5	5.5	N/A
Chemical purity (particulates):	Clear and colorless; particle-free	6 h	Clear, colorless, particle free	Clear, colorless, particle free	Clear, colorless, particle free
		8 h	Clear, colorless, particle free	Clear, colorless, particle free	N/A

## Discussion

[^18^F]FDG-PET/CT is the current preferred method for evaluating multiple myeloma response to therapy however there is a possibility of both false-negatives and false positives [32–36]. Multiple studies have reported myeloma with a low expression of hexokinase-2 which results in false negative at rates of up to 12% [32, 33]. False positives have been reported due to infection, osteonecrosis, and changes due to radiation therapy [34–36]. This highlights a need for a more specific PET tracer to monitor treatment and predict therapy efficacy.

Isatuximab was the natural choice for exploration due to its recent approval by the FDA for multiple myeloma treatment. Our results have shown that [^89^Zr]Zr-DFO-Isatuximab is highly selective agent capable of imaging multiple myeloma with results similar to previous reports [16, 17]. Treatments of Isatuximab plus Pomalidomide have been approved by the FDA for patients with refractory multiple myeloma. In Daratumumab refractory patients, Isatuximab + Pomalidomide has been reported to have a 14% overall response rate and a clinical benefit rate of 42.9% [20]. [^89^Zr]Zr-DFO-Isatuximab may be used on its own or in combination with either Pomalidomide or Ricolinostat to increase image quality or mimic treatment conditions [22, 37]. Additionally, since Isatuximab does not cause shedding of CD38 (as Daratumumab does), [^89^Zr]Zr-DFO-Isatuximab imaging could be used to investigate response to not only Isatuximab treatment, but other CD38 targeting antibodies [9].

A limitation of our study is the high variance in [^89^Zr]Zr-DFO-Isatuximab tumor uptake in the OPM2 xenografts. This may be a result of the heterogeneity of CD38 expression in the tumor and should be further investigated. Previous studies have shown inhibition of CD38 enzymatic activity with Isatuximab. *In vitro* studies observing this were performed at 30 µg/mL whereas our *in vitro* studies used concentrations of 16 ng/mL [38]. Previous *in vivo* studies similarly also used up to 25 µg/mouse and did not note any enzymatic inhibition [16]. Production of a clinical dose of [^89^Zr]Zr-DFO-Isatuximab was similar to other clinically available radiolabeled antibodies with robust radiochemistry and QC procedures. Future studies involving translation into clinical imaging trials are warranted.

## Conclusions

[^89^Zr]Zr-DFO-Isatuximab was prepared and illustrated high binding to CD38 positive cells. Enhancement of [^89^Zr]Zr-DFO-Isatuximab uptake consistent with upregulation of CD38 with Pomalidomide or Ricolinostat was observed in both OPM2 and MM.1S cell lines but not in the U266 control cell line. Dosimetry data for [^89^Zr]Zr-DFO-Isatuximab was collected, analyzed, and found to be similar to other antibodies in the literature. The highest doses were found to be in the routes of clearance (kidney, colon, liver) and the reproductive organs (testes, ovaries). GMP grade [^89^Zr]Zr-DFO-Isatuximab was produced and passed all predetermined quality control criteria with a stability of 6 h at room temperature. Overall, the study suggests that [^89^Zr]Zr-DFO-Isatuximab has favorable properties for imaging CD38 and can be prepared in a form suitable for human use.

## Supplementary Information

Below is the link to the electronic supplementary material.

Supplementary file1 (DOCX 108 KB)

## Data Availability

Data is available from the corresponding author upon request.
