# Supplementary material for: PET Imaging of CD38 and IND enabling studies of [89Zr]Zr-DFO-Isatuximab
Source: Mol Imaging Biol. 2025 Dec 9;28(1):49–59. doi: 10.1007/s11307-025-02062-9 (PMC12966236; doi:10.1007/s11307-025-02062-9)
Supplement: Supplementary file 1 — Supplementary file1 (DOCX 108 KB) [file 11307_2025_2062_MOESM1_ESM.docx]

Figure S1. ^89^Zr-Isatuximab stability results summary

**
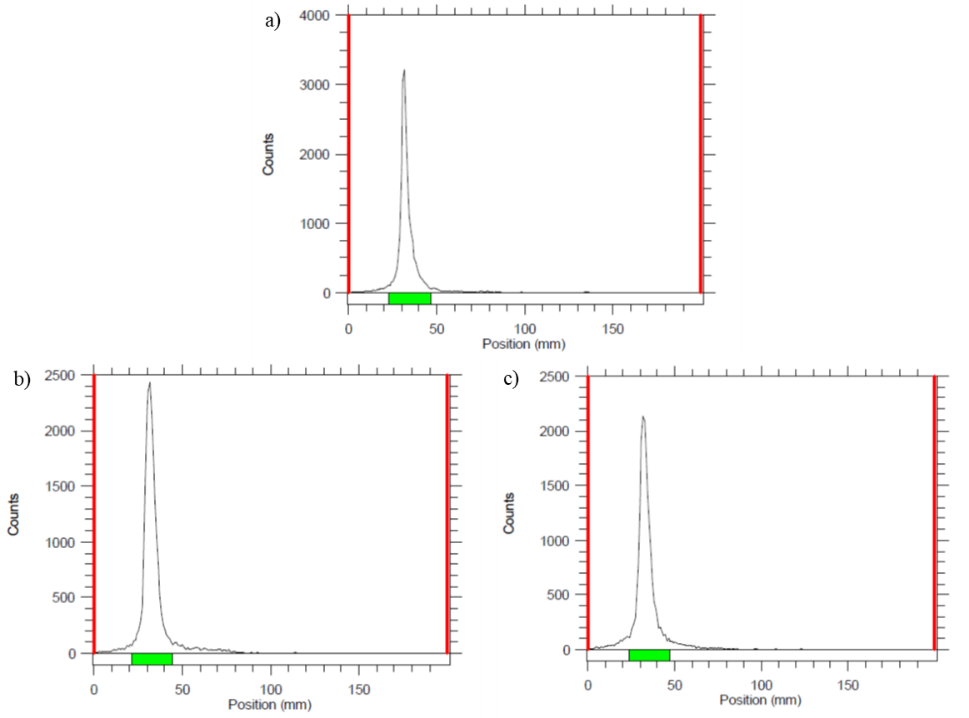
**

Figure S2. ^89^Zr-Isatuximab iTLC 7d stability results in a) 0.9% saline, b) mouse serum, and c) human serum.

Figure S3. Immunoreactivity results for DFO-Isatuximab with a 16:1 molar ratio.
